# Numerical Study of Entropy Production in a Fluidic Oscillator

**DOI:** 10.3390/e28040437

**Published:** 2026-04-13

**Authors:** José Omar Dávalos, Delfino Cornejo-Monroy, Alfredo Villanueva-Montellano, Diana Ortiz-Muñoz, David Luviano-Cruz

**Affiliations:** Instituto de Ingeniería y Tecnología, Universidad Autónoma de Ciudad Juárez, Ciudad Juárez 32310, Chihuahua, Mexico; jose.davalos@uacj.mx (J.O.D.); delfino.cornejo@uacj.mx (D.C.-M.); alfredo.villanueva@uacj.mx (A.V.-M.); diana.ortiz@uacj.mx (D.O.-M.)

**Keywords:** fluidic oscillator, local entropy production rate (LEPR), CFD

## Abstract

A numerical study was conducted to quantify the entropy generation in a fluidic oscillator operating at Reynolds numbers of 30,000, 40,000, and 50,000. Both the local entropy production rate and total entropy were calculated under these operating conditions. Transient computational fluid dynamics (CFD) simulations were carried out using the k−ω shear stress transport (SST) turbulence model. The total entropy was compared with the pressure and driving-force coefficients to establish its relationship with force dynamics. The total entropy showed a periodic evolution synchronized with the jet switching process, while its amplitude increased with Reynolds number and showed a slight phase delay. The pressure and driving-force coefficients exhibited weak fluctuations at the end and beginning of each oscillation period, matching the secondary peaks in total entropy and indicating that these variations arise from residual dissipative effects linked to the jet reattachment stages. The local entropy production rate was concentrated near the feedback channels, Coanda surfaces, and the interaction zone where the jet from the inlet nozzle met the returning flow from the feedback channels. Regions of elevated entropy were detected at the outlet corners due to expansion and pressure drop. The high-velocity jet core exhibited minimal entropy, which increased toward the flanks as the flow decelerated. The results show that entropy generation follows the jet switching motion, reflecting the variations in viscous dissipation and flow dynamics inside the oscillator.

## 1. Introduction

Fluidic oscillators are devices designed to produce periodic changes in fluid flow, forming a consistent oscillatory pattern. This behavior results from fluid dynamics, where the interaction of pressure and inertial forces generates cycles of acceleration, deceleration, and directional changes. Since fluidic oscillators have no moving parts, they are well suited for applications such as flow separation control [1,2,3], enhancement of heat transfer [4,5], and the improvement in aerodynamic performance [6], including drag-reduction strategies under co-flow conditions relevant to aerospace applications [7]. The highly unsteady nature of the internal flow, characterized by complex vortex recirculation and wall interactions, inevitably leads to significant energy degradation, which challenges the overall efficiency of these fluidic systems.

Extensive research has focused on characterizing the internal flow structures to optimize performance. Experimental and numerical studies have documented the formation of recirculation bubbles in the mixing chamber and their role in the feedback mechanism as well as regions of elevated turbulent kinetic energy, typically occurring in areas of strong velocity gradients [8,9,10]. Baghaei and Bergada [11] analyzed the influence of flow conditions on the internal flow structures of a fluidic oscillator. They performed computational fluid dynamics (CFD) calculations by varying the Reynolds number (Re) and observed that this parameter affected both the oscillation frequency and its amplitude. Their results showed that, as Re increased, the flow became more unstable and deviated from a linear frequency −Re relationship. Jabbari and Smaeili [12] numerically analyzed the flow dynamics inside optimized fluidic oscillators. They found that increasing Re modifies both the harmonic and subharmonic content of the oscillating jet, indicating a transition toward a chaotic regime. Using three-dimensional unsteady flow simulations together with experimental measurements, Chen et al. [13] investigated the flow characteristics of a sweeping jet generated by a 10 mm throat-curved fluidic oscillator with different effective chamber lengths. They found that significant variations in effective length modify the internal flow structure by weakening the separation vortex and shifting the jet attachment point within the mixing chamber, which alters the relationship between bulk velocity and oscillation frequency and, under extreme conditions, can even suppress jet oscillation. Extending the discussion on geometric modifications and their impact on the internal flow field, Hussain et al. [14] analyzed the performance of a fluidic oscillator by varying the number of ribs on the Coanda surface. They found that an optimal configuration with four ribs reduced the overall pressure drop by more than 50%, leading to improved jet stability. They also reported that an excessive number of ribs increased flow resistance and energy dissipation, mainly due to intensified vortex shedding and chaotic pressure fluctuations. Guy et al. [15] studied numerically the effect of feedback channel length on the internal flow behavior of a fluidic oscillator. Their results showed that extending the feedback channel modifies the pressure gradient and unsteady flow conditions inside the mixing chamber, which changes the jet dynamics and induces transitions between low-frequency, high-amplitude and high-frequency, low-amplitude oscillatory modes. Complementary to the pressure loss analysis, the driving-force coefficient, $C_{d}$, quantifies the instantaneous pressure imbalance between the lateral feedback channels that induces the feedback flow in fluidic oscillators. As defined by Loe et al. [16], $C_{d}$ is expressed as a normalized pressure difference, which represents the effective forcing mechanism responsible for directing momentum within the device. This pressure-driven force governs the unsteady redistribution of momentum that sustains the oscillation process and directly influences viscous dissipation and overall energy losses in the system.

As discussed in previous studies, the internal flow behavior of fluidic oscillators is governed by a strong coupling between the flow regime, the development of internal flow structures, and geometry. This coupling controls the evolution of pressure and velocity fields, as well as the formation and switching of coherent flow structures that characterize oscillatory behavior. Within these complex interactions, a portion of the mechanical energy supplied to the flow is continuously dissipated through viscous effects, leading to pressure losses and reduced performance [17,18,19].

These viscous and energy losses may be interpreted from a thermodynamic perspective, where the degradation of mechanical energy is evaluated in terms of entropy generation. Herwig and Schmandt [20] suggest that single-valued magnitudes used to evaluate performance and efficiency, such as aerodynamic coefficients and pressure drops, can be replaced by field information on fluid dissipation obtained through entropy production. They concluded that entropy generation is the key factor associated with irreversible energy degradation in the flow. Entropy arises from viscous and thermal dissipation. Viscous dissipation refers to the loss of mechanical energy due to the internal friction generated by fluid viscosity, converting part of the kinetic energy into internal energy, significantly affecting the flow, particularly in regions with high-velocity gradients [21].

Zhao et al. [22] analyzed the entropy production in a centrifugal pump under different viscosity conditions. The influence of viscosity on the distribution of entropy production was numerically investigated. Also, they found that total entropy production increased with viscosity. Turbulent dissipation was greater at low viscosity, whereas time-averaged dissipation dominated at high viscosity. In oscillatory flow configurations, entropy production has been previously discussed in relation to non-stationary dynamics and flow dissipation mechanisms [23]. However, these studies are not directly associated with fluidic oscillator devices, where self-sustained oscillations arise from purely geometric feedback mechanisms, and do not provide a detailed quantification of viscous entropy generation during the oscillation cycle. This highlights the need to investigate entropy production in fluidic oscillators, where oscillatory dynamics are closely linked to flow dissipation. In this work, transient three-dimensional CFD simulations were performed to analyze entropy production within an angled fluidic oscillator. The study was conducted at Re = 30,000, 40,000, and 50,000. The total entropy of the system was calculated during the oscillation periods and correlated with both pressure losses and the driving-force coefficient. In addition, velocity contours were obtained and compared with local entropy production rate (LEPR) contours, providing complementary insight into flow structures and their relation to regions of elevated LEPR. This work provides a thermodynamic perspective on fluidic oscillator performance by linking local viscous entropy production to oscillatory flow dynamics and pressure losses.

## 2. Governing Equations

For a Newtonian, incompressible, and turbulent flow, the governing equations are given by the Reynolds-Averaged Navier–Stokes formulation through the continuity and momentum equations [24]:

(1) $$\frac{\partial {\bar{u}}_{i}}{\partial x_{i}}=0$$

(2) $$\frac{\partial {\bar{u}}_{i}}{\partial t}+\bar{u_{j}}\frac{\partial {\bar{u}}_{i}}{\partial x_{j}}=-\frac{1}{\rho }\frac{\partial \bar{p}}{\partial x_{i}}+\frac{\partial }{\partial x_{i}}\left[\nu \left(\frac{\partial {\bar{u}}_{i}}{\partial x_{j}}+\frac{\partial {\bar{u}}_{j}}{\partial x_{i}}\right)-\frac{\partial \bar{u_{i}^{\prime }u_{j}^{\prime }}}{\partial x_{j}}\right]$$

where ρ is the fluid density, ν is the kinematic viscosity, $\bar{p}$ is the mean pressure, and ${\bar{u}}_{i}$ represents the time-averaged velocity components (i,j=1,2,3). Although the incompressible continuity equation does not contain an explicit temporal derivative due to constant density, it is solved at every time step alongside the momentum equations. In Equation (2), the Reynolds stress term, $\bar{u_{i}^{\prime }u_{j}^{\prime }}$, accounts for turbulent fluctuations and is modeled using the Shear Stress Transport (SST) k−ω turbulence model [25]. The transport equations for the turbulent kinetic energy (k) and the specific dissipation rate (ω) are given by

(3) $$\frac{\partial k}{\partial t}+{\bar{u}}_{j}\frac{\partial k}{\partial x_{j}}=\frac{\partial }{\partial x_{j}}\left[\left(\nu +\sigma_{k}\nu_{t}\right)\frac{\partial k}{\partial x_{j}}\right]+P^{k}-\beta^{*}k\omega$$

(4) $$\frac{\partial \omega }{\partial t}+{\bar{u}}_{j}\frac{\partial \omega }{\partial x_{j}}=\frac{\partial }{\partial x_{j}}\left[\left(\nu +\sigma_{\omega }\nu_{t}\right)\frac{\partial \omega }{\partial x_{j}}\right]+\alpha S^{2}-\beta^{*}\omega^{2}+2\left(1-F_{1}\right)\frac{\sigma_{\omega 2}}{\omega }\frac{\partial k}{{\partial x}_{i}}\frac{\partial \omega }{{\partial x}_{i}}$$

Here, k denotes the turbulent kinetic energy and ω represents the specific dissipation rate. The coefficients α, $F_{1}$, ξ, and ${CD}_{\omega }$ required in Equations (3) and (4) are given by Equations (5)–(8):

(5) $$\alpha =F_{1}\alpha_{k-\omega }+\left(1-F_{1}\right)\alpha_{k-\varepsilon }$$

(6) $$F_{1}=tanh\left(\xi^{4}\right)$$

(7) $$\xi =min\left[max\left\{\frac{\sqrt{k}}{\beta^{*}\omega d},\frac{500\nu }{d^{2}\omega }\right\}\right]\frac{4\sigma_{\omega ,k-\varepsilon }k}{CD_{\omega }d^{2}}$$

(8) $$CD_{\omega }=max\left\{\frac{2\sigma_{\omega ,k-\varepsilon }}{\omega }\right\}\frac{\partial k}{{\partial x}_{i}}\frac{\partial \omega }{{\partial x}_{i}},10^{-10}$$

The constants of the turbulence model are $\beta^{*}=0.09$, $\alpha_{k-\omega }=0.556$, $\alpha_{k-\varepsilon }=0.44$, $\sigma_{k}=0.85$, $\sigma_{\omega }=0.5$, $\sigma_{\omega ,\;k-\varepsilon }=0.856$. These constants correspond to the standard SST k–ω model [25].

For turbulent flows, the LEPR due to viscous dissipation can be decomposed into two contributions [26,27], as shown in Equations (9) and (10). A summary of the theoretical background supporting this formulation is provided in Appendix A.

(9) $$\begin{array}{c} {\dot{S}}_{\bar{D}}^{\prime \prime \prime }=\frac{2\mu_{eff}}{T_{ref}}\left[{\left(\frac{\partial {\bar{u}}_{1}}{\partial x_{1}}\right)}^{2}+{\left(\frac{\partial {\bar{u}}_{2}}{\partial x_{2}}\right)}^{2}+{\left(\frac{\partial {\bar{u}}_{3}}{\partial x_{3}}\right)}^{2}\right]\\ +\frac{\mu_{eff}}{T_{ref}}\left[{\left(\frac{\partial {\bar{u}}_{2}}{\partial x_{1}}+\frac{\partial {\bar{u}}_{1}}{\partial x_{2}}\right)}^{2}+{\left(\frac{\partial {\bar{u}}_{3}}{\partial x_{1}}+\frac{\partial {\bar{u}}_{1}}{\partial x_{3}}\right)}^{2}+{\left(\frac{\partial {\bar{u}}_{2}}{\partial x_{3}}+\frac{\partial {\bar{u}}_{3}}{\partial x_{2}}\right)}^{2}\right] \end{array}$$

(10) $$\begin{array}{c} {\dot{S}}_{D^{\prime }}^{\prime \prime \prime }=\frac{2\mu_{eff}}{T_{ref}}\left[{\left(\frac{\partial u_{1}^{\prime }}{\partial x_{1}}\right)}^{2}+{\left(\frac{\partial u_{2}^{\prime }}{\partial x_{2}}\right)}^{2}+{\left(\frac{\partial u_{3}^{\prime }}{\partial x_{3}}\right)}^{2}\right]\\ +\frac{\mu_{eff}}{T_{ref}}\left[{\left(\frac{\partial u_{2}^{\prime }}{\partial x_{1}}+\frac{\partial u_{1}^{\prime }}{\partial x_{2}}\right)}^{2}+{\left(\frac{\partial u_{3}^{\prime }}{\partial x_{1}}+\frac{\partial u_{1}^{\prime }}{\partial x_{3}}\right)}^{2}+{\left(\frac{\partial u_{3}^{\prime }}{\partial x_{2}}+\frac{\partial u_{2}^{\prime }}{\partial x_{3}}\right)}^{2}\right] \end{array}$$

Here, $\mu_{eff}$ is the effective dynamic viscosity, $T_{ref}$ is the reference temperature, while ${\bar{u}}_{i}$ and $u_{i}^{\prime }$ are the time-averaged and turbulent velocity fluctuations, respectively. The directions in the Cartesian coordinate system are denoted by i = 1, 2, and 3, corresponding to the x, y, and z axes.

In the case of Equation (9), CFD approaches such as Reynolds-Averaged Navier–Stokes cannot directly resolve the detailed velocity fluctuations required to compute entropy. A method was proposed in [28,29] to simplify the calculation of ${\dot{S}}_{D^{\prime }}^{\prime \prime \prime }$, depending on the employed turbulence model. For the k−ω SST turbulence model, this approach yields the expression given in Equation (11).

(11) $${\dot{S}}_{D^{\prime }}^{\prime \prime \prime }=\frac{k\omega \rho }{T_{ref}}\beta$$

where β is a constant of the SST turbulence model equal to 0.09 [25], and $T_{ref}$ = 300 [27] is the temperature-assumed constant. The total entropy produced is obtained from the sum of the volume integrals of Equations (9) and (11) over the domain, as shown in Equation (12):

(12) $${\dot{S}}_{D}=\int_{V}{\dot{S}}_{D}^{\prime \prime \prime }dV=\int_{V}{\dot{S}}_{\bar{D}}^{\prime \prime \prime }+\int_{V}{\dot{S}}_{D^{\prime }}^{\prime \prime \prime }$$

The formulation and analysis presented in this study are based on the following assumptions. Fluid properties are treated as uniform, and the flow is considered incompressible. The simulations are performed under isothermal conditions, and therefore entropy production associated with heat transfer is neglected. Under these conditions, density is treated as constant and no equation of state or energy equation is required. As a result, the local entropy production rate is solely attributed to viscous dissipation. Under the operating conditions considered in this study, no external thermal gradients are imposed, and the working fluid remains at nearly uniform temperature, with no significant heating observed. Since the objective of this work is to analyze hydrodynamic irreversibility associated with jet switching dynamics, the isothermal assumption provides a physically consistent framework for isolating viscous entropy production. Turbulence effects are modeled using a Reynolds-averaged approach. The analysis is based on instantaneous flow fields obtained from the transient simulations. This formulation is consistent with the operating conditions of the fluidic oscillator investigated in this study.

## 3. Methodology

### 3.1. Computational Model and Numerical Setup

**Figure 1:**
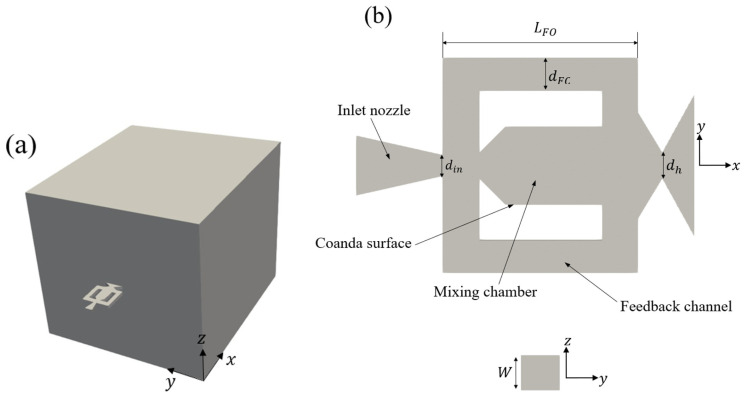
Geometric model of the fluidic oscillator. (**a**) 3D computational domain; (**b**) schematic representation of the domain details.

The fluidic oscillator geometry follows the benchmark design described in [30]. The device is characterized by a hydraulic diameter $d_{h}=25$ mm and a constant aspect ratio based on a width W=25 mm. To prevent boundary influence on the jet discharge and its downstream development, the computational domain extends $60d_{h}$ in the streamwise direction and $50d_{h}$ in the spanwise and vertical directions (Figure 1).

**Figure 2:**
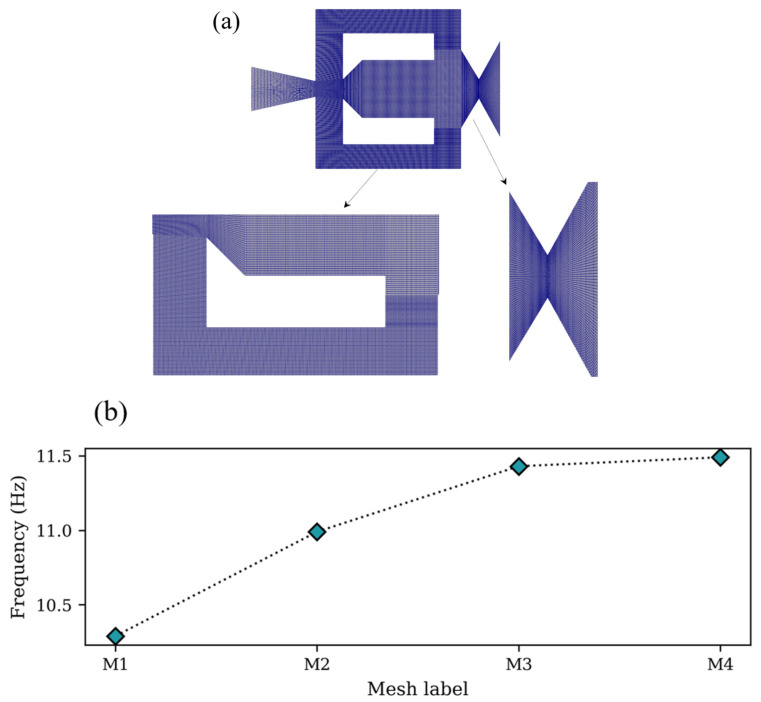
Computational grid and mesh independence analysis. (**a**) Meshed fluidic oscillator geometry. (**b**) Oscillation frequency as a function of mesh density.

The domain was discretized with hexahedral cells using the multiblock technique. The mesh, shown in Figure 2a, consisted of 2,498,000 cells. The number of cells was determined from a mesh convergence analysis comparing four different mesh sizes to ensure mesh-independent results (Figure 2b). The results indicated that the variation in the oscillation frequency between the largest mesh, M4 (3,216,000 cells), and the previous, M3 (2,498,000 cells), was less than 1%. Therefore, mesh M3 was selected as the reference grid for the simulations.

### 3.2. Flow Conditions and Boundary Conditions

The study was performed at three Re numbers, 30,000, 40,000, and 50,000, with $d_{h}$ as the characteristic length. These Reynolds numbers correspond to the fully turbulent operating regime of the fluidic oscillator, where self-sustained oscillations are stable. The selected range is consistent with commonly reported experimental conditions and ensures representative device operation while enabling comparison with previous studies [30]. The Re number is defined by Equation (13), following standard definitions reported in [9,30].

(13) $$Re=\frac{\rho U_{out}d_{h}}{\mu }$$

**Figure 3:**
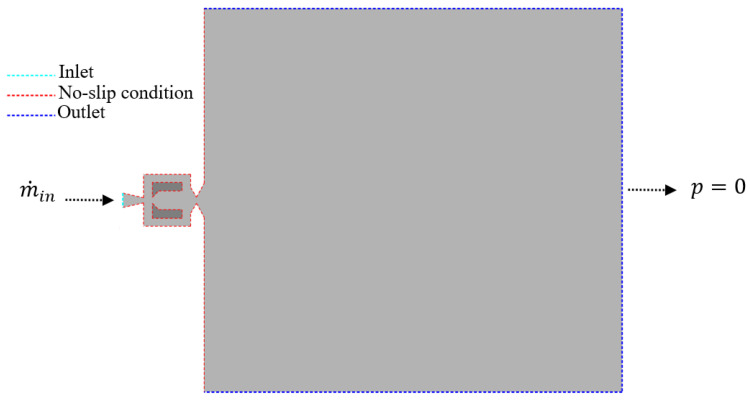
Schematic representation of the computational domain and applied boundary conditions.

A schematic representation of the computational domain and the applied boundary conditions are shown in Figure 3. At the inlet, a mass flow rate boundary condition was imposed. The inlet mass flow rate ${\dot{m}}_{in}$ was determined from the continuity equation as

(14) $${\dot{m}}_{in}=U_{out}A_{out}\rho$$

where ${\dot{m}}_{in}$ is the mass flow rate and $A_{out}$ is the area at the outlet of the fluidic oscillator. At the outlet of the computational domain, a pressure outlet condition was applied, with the static pressure set to zero gauge pressure (p=0). All solid surfaces of the domain, including the sidewalls as well as the upper and lower surfaces, were defined as no-slip walls.

The turbulence was modeled using the k−ω SST approach [25]. This model has been widely validated in previous results [31,32,33]. The coupled scheme was selected for pressure–velocity coupling, and the convergence criterion was set to 1 × 10^−5^ with a maximum of 400 iterations per time step. The time step ∆t = 1 × 10^−4^ s was selected to adequately resolve the oscillatory dynamics. Based on the highest expected oscillation frequency of approximately 18 Hz, this corresponds to 556 time steps per oscillation period, largely exceeding the Nyquist criterion and ensuring sufficient temporal resolution of the jet switching process and the associated entropy production.

### 3.3. Flow Analysis Parameters

Based on Equations (10) and (11), a field variable was implemented in the ANSYS Fluent 2025 CFD solver to compute the LEPR at each time step during the transient simulations. The LEPR was then spatially integrated over the domain to obtain the total entropy production. This quantity was compared with both the pressure coefficient and the driving-force coefficient, defined in Equations (15) and (16), following [16]:

(15) $$C_{p}=\frac{P_{t0}-P_{t1}}{q_{0}}$$

(16) $$C_{d}=\frac{2\left(\frac{P_{t3}-P_{t2}}{q_{0}}\right)d_{i}}{d}$$

where $P_{t0}$ is the total pressure at the inlet of the fluidic oscillator, $P_{t1}$ is the total pressure at the outlet, $P_{t2}$ and $P_{t3}$ are the total pressures at the inlets on each side of the feedback channels, and $q_{0}$ is the dynamic pressure at the inlet of the fluidic oscillator, whereas d and $d_{i}$ represent the inlet nozzle and feedback channel widths, respectively.

## 4. Results

**Figure 4:**
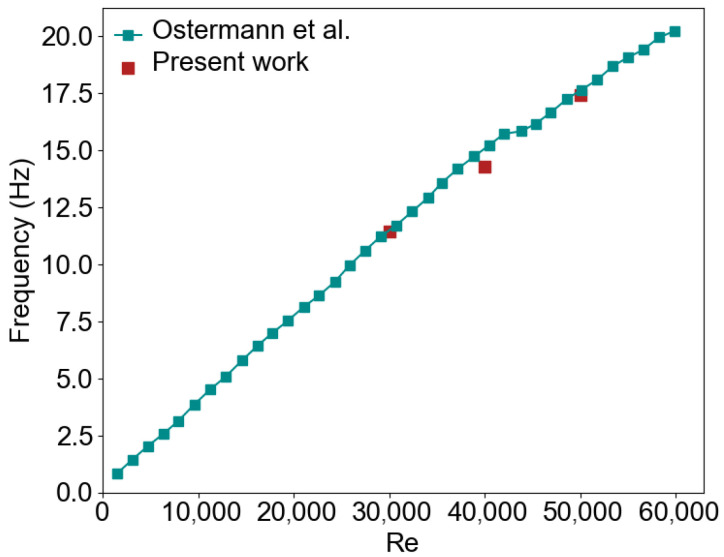
Comparison of the predicted oscillation frequencies with experimental measurements reported by Ostermann et al. [30].

Figure 4 shows the comparison between the CFD predictions and experimental results from [30]. Experimental measurements of jet switching frequency were reported for a geometrically similar fluidic oscillator operating within a comparable Reynolds number range, providing benchmark data for validation. The reference study reported a nearly linear increase in switching frequency with Reynolds number and showed that the oscillation mechanism is governed by volume transport through the feedback channels. The predicted frequency exhibits good agreement with the experimental frequencies. At Re = 40,000, a slight deviation is observed, but the difference from the experimental value remains below 5%. The frequencies at Re = 30,000 and Re = 50,000 agree closely with those reported by Ostermann et al. [30], with deviations lower than 1%. These results confirm the validity of the numerical model employed in this study.

**Figure 5:**
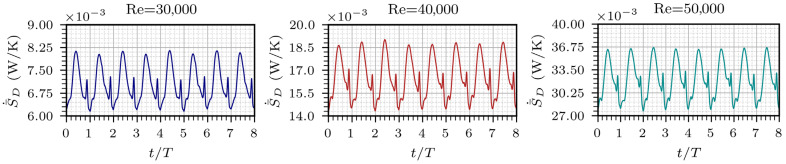
Temporal evolution of the total entropy generation rate over eight oscillation cycles for different Reynolds numbers.

Figure 5 presents the total entropy generated over eight cycles. The magnitude of entropy oscillations increases with Re. The amplitude of the entropy peaks increases by a factor of 2.3 between Re = 30,000 and 40,000, and by 1.94 between 40,000 and 50,000. A critical finding is the phase lag in the entropy peak relative to the oscillation period (T). At Re=30,000, the maximum dissipation occurs at t≈ 0.4*T*. However, as inertia increases at Re=50,000, this peak shifts to t≈ 0.45*T*. This behavior is attributed to the increase in inertial forces, which results in a temporal shift in the viscous dissipation effects. A secondary peak of lower magnitude is also observed near the end of the period, around 0.9*T*. The ratios between the lower and higher entropy peaks are 0.44, 0.30, and 0.23 for Re = 30,000, 40,000, and 50,000, respectively. The secondary peak exhibits reduced magnitude at higher Re and the energy losses decrease before the start of a new cycle. However, the dissipative effects increase around the middle of the period. Another subtle fluctuation is detected near 0.1*T*, at the initial stage of the cycle, becoming more pronounced and slightly sharper with higher Re, although the variation in magnitude remains minimal. These fluctuations can be attributed to the residual recirculation and shear effects caused by the jet wall attachment, leading to a more pronounced variation at the end of a cycle and weaker ones at the beginning. The dissipation losses are periodic and governed by the oscillation mechanism. While the oscillation mechanism is not affected by Re, the magnitude and evolution of entropy are influenced by the increased turbulence. While the present results clearly show an increase in entropy peak amplitude with Reynolds number, the identification of a specific scaling law or an asymptotic saturation behavior at higher Reynolds numbers is left for future investigation.

**Figure 6:**
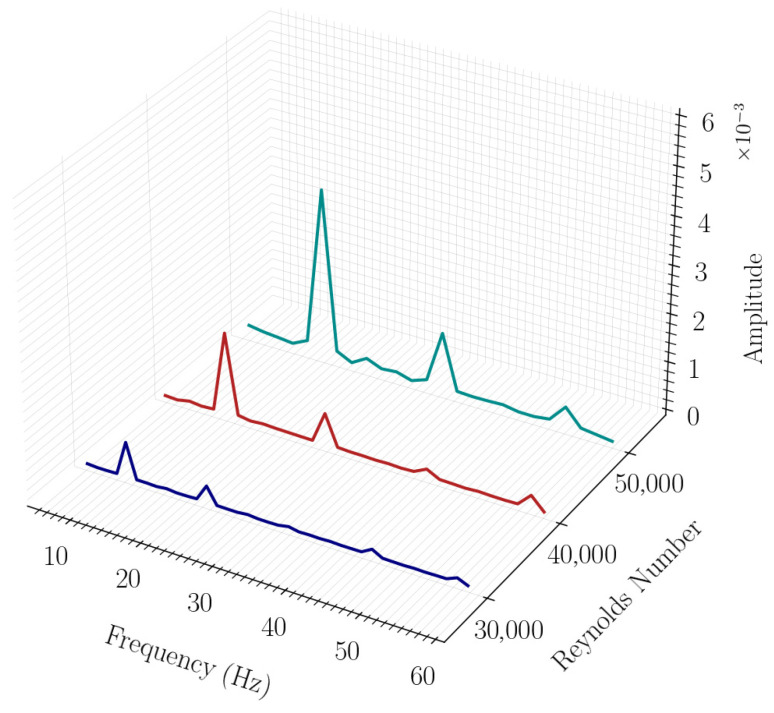
Comparison of frequency spectra of total entropy for different Reynolds numbers.

The spectral characteristics of the total entropy signal are presented in Figure 6. The dominant frequencies (11.43 Hz, 14.29 Hz, and 17.43 Hz for increasing Re) align closely with the fluidic oscillator frequencies, indicating that viscous entropy production is directly modulated by the jet switching process. Additional spectral peaks appear at integer multiples of the dominant frequency, reflecting periodic features associated with jet deflection and recirculation within the oscillator. As Reynolds number increases, the amplitude of these higher-frequency components becomes more pronounced, indicating stronger fluctuations and enhanced viscous dissipation while preserving the fundamental oscillatory behavior imposed by the geometric feedback mechanism.

**Figure 7:**
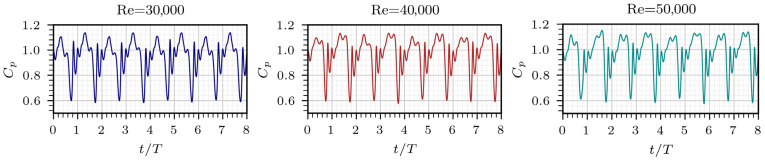
Temporal evolution of pressure coefficient over eight oscillation cycles for different Reynolds numbers.

The pressure coefficient $C_{p}$ at different Re is presented in Figure 7. Even though the values of $C_{p}$ remain nearly constant in all cases due to dynamic pressure scaling, a remarkable difference is observed in its behavior at the largest peak. At Re = 40,000 and 50,000, a split-peak feature occurs, which is not present at Re = 30,000. The entropy peaks presented in Figure 5 coincide with the time periods in which $C_{p}$ fluctuates. At Re = 30,000, the greatest $C_{p}$ peak occurs at 0.3*T* and then decreases; however, a small increment appears between 0.45*T* and 0.5*T*, followed by a subsequent decay. A similar trend is observed at Re = 40,000 and 50,000, which differs as a second peak emerges around 0.45*T*. At the transition between consecutive cycles, noticeable variations in $C_{p}$ are observed. This behavior occurs nearly simultaneously with the secondary entropy peak, indicating a temporal correspondence with the dissipative mechanisms of the flow.

**Figure 8:**
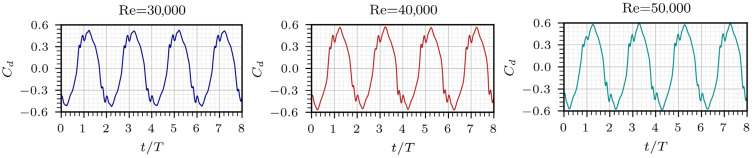
Temporal evolution of load coefficient over eight oscillation cycles for different Reynolds numbers.

The values of $C_{d}$ are presented in Figure 8. The peak-to-peak magnitude of $C_{d}$ shows a slight increase with Re, since the higher inertia relative to viscous effects enhances jet attachment to the sidewalls and intensifies the lateral driving forces. The coefficient $C_{d}$ exhibits one oscillation for every two observed in total entropy and $C_{p}$ within the same time span. This occurs because $C_{d}$ completes a full cycle after two jet-switch events. In all cases, $C_{d}$ showed no noticeable variation between 0.4*T* and 0.45*T*, since the lateral pressure is nearly balanced during the transition. Instead, fluctuations appear at the end and beginning of each cycle when the lateral momentum is stronger, remaining temporally consistent with the secondary entropy peaks. This temporal consistency between the fluctuations of $C_{d}$ and the total entropy peaks highlights the dynamic coupling between lateral momentum redistribution and viscous dissipation during the oscillation cycle, rather than implying a direct pointwise comparison between these quantities.

**Figure 9:**
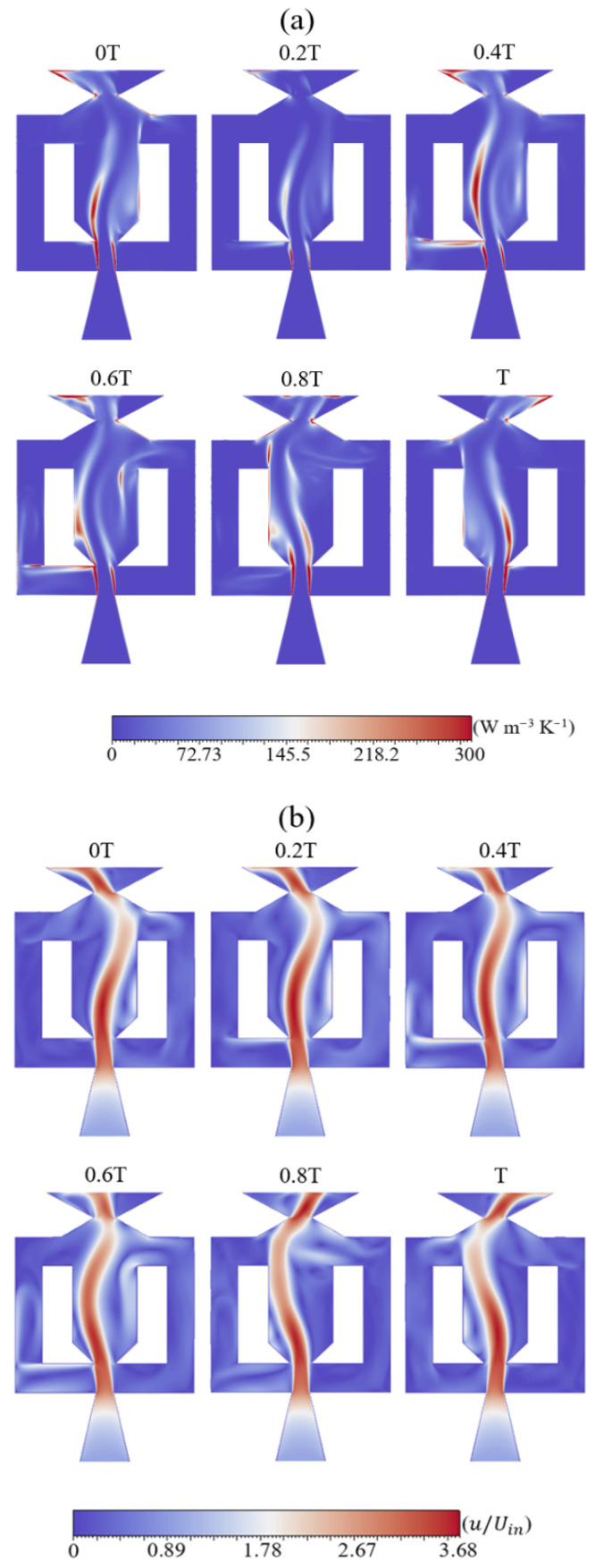
Comparison contours at different time instants within one oscillation cycle of (**a**) LEPR and (**b**) normalized velocity.

Contour plots in Figure 9 show the relationship between LEPR and velocity magnitude. Only Re = 50,000 is presented, as all Re cases exhibit comparable qualitative behavior. In Figure 9a, a localized maximum appears where the jet inlet interacts with the flow from the feedback channels. This entropy concentration is not evenly distributed within the interaction zone. At 0*T*, the entropy is higher on one side of this region and persists until 0.6*T*. At 0.8*T*, entropy becomes more uniform. Toward the end of the period, the entropy level decreases on the initially dominant side while increasing on the opposite one. Entropy accumulation results from the velocity difference between the flow discharged from the inlet nozzle and that returning from the feedback channel. This interaction induces velocity gradients that enhance viscous dissipation. At the mixing chamber, entropy magnitude is lower in comparison with other regions but more broadly distributed. In Figure 9b, the highest velocity follows the jet centerline extending through the chamber, where entropy production remains minimal along the core region. In contrast, entropy increases toward the jet flanks, where velocity gradients are stronger and viscous dissipation is enhanced. Accordingly, the LEPR attains minimum values in regions of high velocity, while entropy accumulation occurs in zones where the flow decelerates. This behavior results from energy losses at the flanks, which induce entropy generation, whereas energy is largely preserved in the core. It is observed that as the jet approaches the left wall of the mixing chamber, the entropy remains nearly constant. Due to the transition to the right side, at 0.6*T*, the jet contacts the wall with minimal influence on the development of entropy. However, near the entrance of the right feedback channel, a localized entropy concentration is detected, which is caused by energy losses. A recirculation entropy bubble grows near the right wall from 0*T* to 0.6*T* and then detaches. This matches the velocity contours, showing that the bubble forms in the same low-velocity region. At 0.8T, the bubble dissipates and reforms at the left wall at the end of the period (T). At the exit nozzle, the sweeping jet from the mixing chamber produces regions of elevated entropy. As the jet moves toward the opposite end of the exit section, the location of higher entropy shifts accordingly in the same direction. As observed in the mixing chamber, the entropy at the exit remains negligible at the jet core, while it increases toward the sides due to energy losses. Entropy concentration occurs at the exit nozzle corners and where the jet discharges into the external flow field. This localized increase in entropy originates from wall-induced viscous dissipation at the corners. The expansion caused by the pressure drop at the outlet also contributes to the intensification of entropy generation. The entropy fields indicate that the entropy evolution is closely linked to the jet motion. The total entropy reaches its maximum during the jet switching stage, where flow separation, reattachment, mixing, and strong shear combine to produce maximum viscous dissipation.

**Figure 10:**
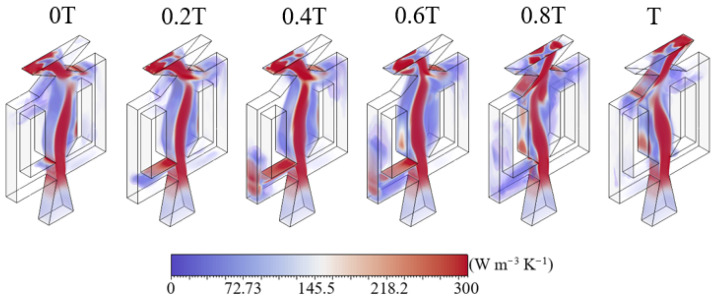
Volume-rendered distribution of LEPR at different time instants within one oscillation cycle.

A volumetric visualization of the LEPR is presented in Figure 10, providing insight into the redistribution of dissipation over an oscillation period. Compared to the mid-plane contours, entropy increases significantly near the upper and lower walls due to reduced velocity and dominant friction along boundaries. This entropy distribution closely follows the jet shape. At the walls of the feedback channels, entropy increases between 0*T* and 0.6*T*, then decays before the cycle restarts. This behavior results from the jet switching induced by the Coanda effect. When the jet attaches to the walls of the feedback channels, viscous dissipation increases in the near-wall region, producing enhanced LEPR. At 0*T*, locally intensified LEPR appears near one of the Coanda surfaces and gradually dissipates over time. As the jet switches sides, a localized region of LEPR begins to emerge on the opposite Coanda surface, becoming more pronounced as the oscillation progresses. At the beginning of a new period, the LEPR region shifts entirely to the Coanda surface, reflecting the transition associated with jet attachment and detachment. At the outlet, the flow undergoes sudden expansion due to the loss of confinement, which produces a pressure drop. As the jet discharges into the external flow field, velocity gradients intensify near the nozzle edges, leading to elevated dissipation. The entropy distribution at the exit is non-uniform, with different intensities between the upper and lower sides, indicating an asymmetric dissipation pattern.

**Figure 11:**
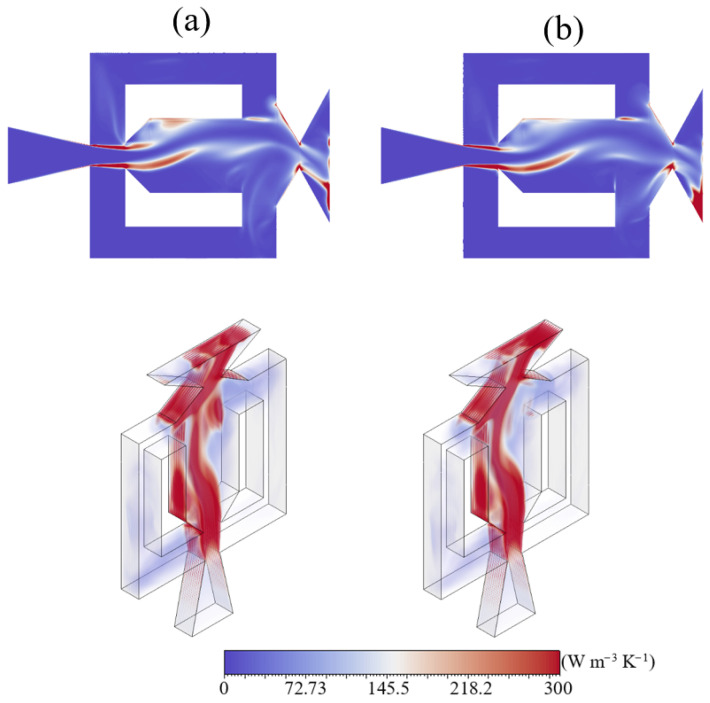
Planar and volumetric LEPR fields near the end of the oscillation cycle: (**a**) at the onset of the increase toward the secondary entropy peak and (**b**) at the secondary entropy peak.

In Figure 11, planar and volumetric LEPR fields are shown at the instant corresponding to the change in entropy trend, marking the onset of the increase toward the secondary peak (Figure 11a), and at the corresponding secondary entropy peak (Figure 11b), with both occurring near the end of the oscillation cycle. During the stage preceding the secondary peak, increased entropy is mainly concentrated along the Coanda surface. In contrast, at the instant of the secondary peak, a slight increase in entropy is observed around the jet core. Additionally, planar contours reveal more pronounced localized regions of increased entropy near the outlet corners at this stage, indicating an enhanced contribution of these regions to the overall entropy production near the end of the oscillation cycle. The volumetric contours provide additional insight into the spatial redistribution of entropy during this stage of the oscillation cycle. Figure 11a shows that regions of elevated entropy remain relatively confined and localized within limited portions of the flow field. In contrast, at the instant of the secondary peak, the entropy field exhibits a broader volumetric spread, with intensified regions extending across the mixing chamber and into the outlet region. This indicates that the secondary peak is not associated with a localized intensification along a single flow structure, but rather with a spatial expansion of dissipative regions involving multiple zones of the internal flow near the end of the oscillation cycle.

It should be noted that the present analysis assumes isothermal flow conditions, which are representative of the subsonic operating regime considered in this study. Under these conditions, entropy production is dominated by viscous dissipation, while thermal effects are negligible. In applications involving higher velocities or significant temperature gradients, additional entropy generation mechanisms associated with heat transfer may become relevant and should be considered in future studies.

## 5. Conclusions

In this paper, CFD computations were carried out to calculate the LEPR and total entropy generated inside a fluidic oscillator. The numerical simulations were performed at Re = 30,000, 40,000, and 50,000. The total entropy exhibits clear oscillatory behavior, with its amplitude increasing by a factor of approximately 2.3 between Re = 30,000 and 40,000, and by 1.94 between Re = 40,000 and 50,000, indicating a reduced growth rate at higher Reynolds numbers.

The phase of the maximum entropy peak shifts from 0.4*T* to 0.45*T* as Reynolds number increases, reflecting enhanced inertial effects. Secondary entropy peaks occur during cycle transitions and coincide with fluctuations in pressure and driving-force coefficients, confirming strong temporal coupling between entropy production and flow switching. The increasing Reynolds number also leads to higher oscillation frequencies and more rapidly varying dissipative dynamics.

Spatially, LEPR is inversely correlated with jet velocity, reaching minimum values along the jet core and increasing toward the jet flanks. Elevated entropy production regions are consistently located along feedback channel walls, the Coanda surface, jet–feedback interaction zones, and outlet nozzle corners.

These results provide a quantitative description of entropy generation mechanisms in fluidic oscillators and demonstrate how Reynolds number controls both the temporal evolution and spatial localization of dissipation. The identified regions of elevated entropy production offer clear guidance for potential geometric modifications, enabling targeted design strategies aimed at reducing dissipative losses. Future work will focus on using these entropy maps to guide geometry modifications across the entire oscillator, aiming to reduce dissipative losses and improve overall performance.

## Data Availability

The data presented in this study are available on request from the corresponding author.

## Nomenclature

$A_{out}$	Outlet area of the fluidic oscillator [m^2^]
$C_{p}$	Pressure coefficient [-]
$C_{d}$	Driving-force coefficient [-]
d	Inlet nozzle width [mm]
$d_{h}$	Hydraulic diameter [mm]
$d_{i}$	Feedback channel width [mm]
$\frac{D}{Dt}$	Material derivative [-]
$f_{i}$	Body force [N m^−3^]
k	Turbulent kinetic energy [m^2^ s^−2^]
$\dot{m_{in}}$	Inlet mass flow rate [kg s^−1^]
p	Static pressure [Pa]
$\bar{p}$	Mean pressure [Pa]
$P_{k}$	Production of turbulent kinetic energy [m^2^ s^−3^]
$P_{ij}^{\prime }$	Viscous stress tensor [Pa]
$q_{0}$	Dynamic pressure at the inlet [Pa]
$\dot{Q}$	Energy transfer rate [W]
Re	Reynolds number [-]
$\dot{S_{D}}$	Total entropy generation rate [W K^−1^]
${\dot{S}}_{D}^{\prime \prime \prime }$	Local entropy production rate [W m^−3^ K^−1^]
t	Time [s]
T	Oscillation period [s]
$\bar{u_{i}}$	Time-averaged velocity component [m s^−1^]
$u_{i}^{\prime }$	Velocity fluctuation [m s^−1^]
$U_{out}$	Outlet velocity [m s^−1^]
W	Oscillator width [mm]
α	Turbulence model coefficient [-]
$\beta ,\beta^{*}$	SST turbulence model constants [-]
$\delta_{ij}$	Kronecker delta [-]
$\varepsilon_{ij}$	Strain-rate tensor [s^−1^]
μ	Dynamic viscosity [Pa s]
$\mu_{eff}$	Effective dynamic viscosity [Pa s]
ν	Kinematic viscosity [m^2^ s^−1^]
$\nu_{t}$	Turbulent kinematic viscosity [m^2^ s^−1^]
ρ	Density [kg m^−3^]
$\sigma_{k}$	Turbulent Prandtl number for k [-]
$\sigma_{\omega }$	Turbulent Prandtl number for ω [-]
Φ	Viscous dissipation function [W m^−3^]
ω	Specific dissipation rate [s^−1^]

## Abbreviations

The following abbreviations are used in this manuscript:

LEPR	Local entropy production rate
CFD	Computational fluid dynamics
SST	Shear stress transport

## Appendix A

For completeness, this appendix summarizes the theoretical background supporting the formulation used in the present analysis. Although the numerical simulations in this work are performed under an incompressible Reynolds-averaged framework, the derivation of viscous dissipation and entropy production is first introduced from the general momentum equation and then specialized to the incompressible case considered here. The derivation begins with the general Navier–Stokes momentum equation in Cartesian coordinates [34]:

(A1) $$\rho \frac{Du_{i}}{Dt}=\rho f_{i}-\frac{\partial p}{\partial x_{i}}+\frac{\partial }{\partial x_{i}}\left(P_{ij}^{\prime }\right)$$

where

(A2) $$P_{ij}^{\prime }=\left(\mu^{\prime }-\frac{2}{3}\mu \right)div\vec{V}\delta_{ij}+2\mu \varepsilon_{ij}=\left(\mu^{\prime }-\frac{2}{3}\mu \right)\frac{\partial u_{i}}{\partial x_{i}}\delta_{ij}+\left[\mu \left(\frac{\partial u_{i}}{\partial x_{j}}+\frac{\partial u_{j}}{\partial x_{i}}\right)\right]$$

By contracting Equation (A1) with the velocity components $u_{i}$, the kinetic energy equation is obtained as

(A3) $$\frac{D}{Dt}\left(\frac{1}{2}u_{i}u_{i}\right)=f_{i}u_{i}-\frac{1}{\rho }u_{i}\frac{\partial p}{\partial x_{i}}+\frac{1}{\rho }\frac{\partial }{\partial x_{i}}\left(P_{ij}^{\prime }u_{j}\right)-\frac{1}{\rho }P_{ij}^{\prime }\frac{\partial u_{j}}{\partial x_{i}}$$

Equation (A3) governs the evolution of the mechanical energy of the flow. The material derivative of the kinetic energy per unit mass $\frac{D}{Dt}\left(\frac{1}{2}u_{i}u_{i}\right)$ characterizes the temporal change in mechanical energy associated with fluid motion. The remaining terms describe the processes through which energy is transferred, redistributed, or dissipated within the system. The contributions related to body forces and pressure gradients correspond to reversible work performed on the fluid, whereas the remaining terms arise from viscous effects. The viscous contributions play two fundamentally different roles. One term accounts for the redistribution of mechanical energy through viscous stresses, enabling energy exchange between different regions of the flow without altering its mechanical form. The other term is associated with viscous deformation induced by velocity gradients and leads to irreversible losses of mechanical energy. This irreversible mechanism converts mechanical energy into internal energy and constitutes the source of entropy generation, forming the physical basis for the definition of viscous dissipation adopted in this work.

To further analyze the contribution associated with viscous effects in Equation (A3), the term involving the viscous stress tensor and velocity gradients is introduced through the viscous dissipation function:

(A4) $$\Phi =P_{ij}^{\prime }\frac{\partial u_{j}}{\partial x_{i}}$$

Using the expression for the viscous stress tensor given above, the viscous dissipation function Φ can be written as

(A5) $$\Phi =\left[\left(\mu^{\prime }-\frac{2}{3}\mu \right)div\vec{V}\delta_{ij}+2\mu \varepsilon_{ij}\right]\frac{\partial u_{j}}{\partial x_{i}}$$

For an incompressible fluid, the bulk viscosity is set to $\mu^{\prime }=0$, and the viscous dissipation function Φ is expanded using the velocity components $u_{1},u_{2},u_{3}$ as

(A6) $$\begin{array}{c} \Phi =2\mu \left[{\left(\frac{\partial u_{1}}{\partial x_{1}}\right)}^{2}+{\left(\frac{\partial u_{2}}{\partial x_{2}}\right)}^{2}+{\left(\frac{\partial u_{3}}{\partial x_{3}}\right)}^{2}\right]\\ \;\;\;\;\;\;\;\;\;\;\;\;\;\;\;\;\;\;\;\;\;\;\;\;\;\;\;\;\;\;\;\;\;\;\;\;\;\;\;\;\;\;\;\;\;\;\;\;\;\;\;\;\;\;\;\;\;\;\;\;\;\;\;\;\;\;\;\;\;\;\;\;\;\;\;\;\;\;\;\;\;\;\;\;\;\;\;\;\;\;\;\;\;\;\;\;+\mu \left[{\left(\frac{\partial u_{2}}{\partial x_{1}}+\frac{\partial u_{1}}{\partial x_{2}}\right)}^{2}+{\left(\frac{\partial u_{3}}{\partial x_{1}}+\frac{\partial u_{1}}{\partial x_{3}}\right)}^{2}+{\left(\frac{\partial u_{2}}{\partial x_{3}}+\frac{\partial u_{3}}{\partial x_{2}}\right)}^{2}\right]\\ \;\;\;\;\;\;\;\;\;\;\;\;\;\;\;\;\;\;\;\;\;\;\;\;\;\;\;\;\;\;\;\;\;\;\;\;\;\;\;\;\;\;-\frac{2}{3}\mu {\left(\frac{\partial u_{1}}{\partial x_{1}}+\frac{\partial u_{2}}{{\partial x}_{2}}+\frac{\partial u_{3}}{\partial x_{3}}\right)}^{2} \end{array}$$

Here, μ is the dynamic viscossity. Considering the continuity equation, given by

(A7) $$\frac{\partial u_{1}}{\partial x_{1}}+\frac{\partial u_{2}}{{\partial x}_{2}}+\frac{\partial u_{3}}{\partial x_{3}}=0$$

the Equation (A6) simplifies to

(A8) $$\begin{array}{c} \Phi =2\mu \left[{\left(\frac{\partial u_{1}}{\partial x_{1}}\right)}^{2}+{\left(\frac{\partial u_{2}}{\partial x_{2}}\right)}^{2}+{\left(\frac{\partial u_{3}}{\partial x_{3}}\right)}^{2}\right]\\ \;\;\;\;\;\;\;\;\;\;\;\;\;\;\;\;\;\;\;\;\;\;\;\;\;\;\;\;\;\;\;\;\;\;\;\;\;\;\;\;\;\;\;\;\;\;\;\;\;\;\;\;\;\;\;\;\;\;\;\;\;\;\;\;\;\;\;\;\;\;\;\;\;\;\;\;\;\;\;\;\;\;\;\;\;\;\;\;\;\;\;\;\;\;\;\;+\mu \left[{\left(\frac{\partial u_{2}}{\partial x_{1}}+\frac{\partial u_{1}}{\partial x_{2}}\right)}^{2}+{\left(\frac{\partial u_{3}}{\partial x_{1}}+\frac{\partial u_{1}}{\partial x_{3}}\right)}^{2}+{\left(\frac{\partial u_{2}}{\partial x_{3}}+\frac{\partial u_{3}}{\partial x_{2}}\right)}^{2}\right] \end{array}$$

The viscous energy dissipation represents the rate of conversion of mechanical energy into internal energy and can be expressed as an equivalent energy transfer rate, $\Phi =\dot{Q}$. Accordingly, the LEPR is defined as [21,35]

(A9) $${\dot{S}}_{D}^{\prime \prime \prime }=\frac{\dot{Q}}{T_{ref}}=\frac{\Phi }{T_{ref}}$$
